# An integrated genome-wide association analysis on rheumatoid arthritis data

**DOI:** 10.1186/1753-6561-1-s1-s35

**Published:** 2007-12-18

**Authors:** Jun Zhang, Xiaofeng Zhu, Richard S Cooper

**Affiliations:** 1 Department of Statistics, University of Chicago, 5734 South University Avenue, Chicago, Illinois 60637 USA; 2 Department of Epidemiology and Biostatistics, Case Western Reserve University, 2103 Cornell Road, Cleveland, Ohio 44106 USA; 3 Department of Preventive Medicine and Epidemiology, Loyola University, 2160 South First Avenue, Maywood, Illinois 60153 USA

## Abstract

We propose a nonparametric association analysis combining both family and unrelated case-control genotype data. Under the assumption of Hardy-Weinberg equilibrium, we formed an affected group to compare with a group of unaffecteds.

Comparison with traditional case-control chi-square test and transmission-disequilibrium test shows that this new approach has noticeably improved power. All analysis was based on the simulated rheumatoid arthritis data provided by Genetic Analysis Workshop 15. In the situation of population stratification, we also suggest an approach to update the genotype data using principal components. However, the Genetic Analysis Workshop 15 simulation data does not simulate population stratification. All analysis was done without knowledge of the answers.

## Background

Traditional linkage analysis has achieved great success in the genetic dissection of mendelian diseases caused by a single gene with large effect. However, it is well known that association analysis has more power than linkage analysis for complex diseases such as rheumatoid arthritis (RA) [1]. Nowadays genome-wide association studies have been widely planned and carried out due to biotechnical improvements and decreasing experimental costs. Traditional approaches to association study designs are either family-based or unrelated case-control subjects based. Here we demonstrate an integrated association analysis using both family and unrelated simulation data on RA from Genetic Analysis Workshop 15 (GAW15).

## Methods

### Simulated data without population stratification

The RA data set was simulated according to familial patterns and other environmental effects. Each of the 100 replicates has 1500 nuclear families consisting of one affected sibling pair (ASP) and their parents, and 2000 unrelated unaffected individuals as controls. Markers include 730 microsatellite markers, 9187 evenly distributed SNPs on 22 autosomal chromosomes, and 17,820 dense SNPs on chromosome 6. In the analysis, we used the first 200 families and the first 200 people of the 2000 controls. To include unrelated cases in the analysis, we randomly picked one of the two affected siblings from the next 200 families. Our final data set includes 200 families, 200 unrelated cases, and 200 controls. Among the 200 selected families, there were 56 families with a single parent and two families with both parents affected. In the most general setting, we form one group of all affected individuals consisting of affected siblings, affected parents, and unrelated cases, which was compared with a group of all unaffected individuals consisting of unaffected siblings, unaffected parents, and unrelated controls. Depending on the number of affected parents, there are three possible groupings for a family with *r *affected siblings with genotype *x*_1_,..., *x*_*r*_; *s *unaffected siblings with genotype *y*_1_,..., *y*_*s*_, and parents with genotype *x*_*m *_and *x*_*f *_Here, genotypes *x *and *y *denote the number of a particular allele whose allele frequency is *p*. Suppose in the data there are *l *families with both unaffected parents, *m *families with one affected parent (say the mother), *n *families with both affected parents, and additionally unrelated cases *wi*, *i *= 1,..., *u*, and *v *controls *z*_*i*_, *i *= 1,..., *v*. The allele frequencies of the two groups are given by:

$$p_{a}\;=\frac{\sum_{i=1}^{l}(x_{1}^{\left(i\right)}+...+x_{r}^{\left(i\right)})+\sum_{j=1}^{m}\left(x_{1}^{\left(j\right)}+...+x_{r}^{\left(j\right)}+x_{m}^{\left(j\right)}\right)+\sum_{k=1}^{n}(x_{1}^{\left(k\right)}+...+x_{r}^{\left(k\right)}+x_{m}^{\left(k\right)}+x_{f}^{\left(k\right)})+\sum w_{i}}{2\left[lr+m(r+1)+n(r+2)+u\right]}$$

$$p_{u}\;=\frac{\sum_{i=1}^{l}(y_{1}^{\left(i\right)}+...+y_{s}^{\left(i\right)}+x_{m}^{\left(i\right)}+x_{f}^{\left(i\right)})+\sum_{j=1}^{m}\left(y_{1}^{\left(j\right)}+...+y_{s}^{\left(j\right)}+x_{f}^{\left(j\right)}\right)+\sum_{k=1}^{n}(y_{1}^{\left(k\right)}+...+y_{s}^{\left(k\right)})+\sum z_{i}}{2\left[l(s+2)+m(s+1)+ns+v\right]}.$$

We then use a normal test statistic $z=\frac{p_{a}-p_{u}}{\sqrt{Var(p_{a}-p_{u})}}$, which is a generalization of Risch and Teng's result [2]. In particular, *Var*(*p*_*a *_- *p*_*u*_) = *Var*(*p*_*a*_) + *Var*(*p*_*u*_) - 2*Cov*(*p*_*a*_, *p*_*u*_). Assuming Hardy-Weinberg equilibrium, each term is given below:

Var(p)a=l(r2+r)+m(r2+3r+2)+2n(r2+2r+2)+2u4[lr+m(r+1)+n(r+2)+u]2p(1−p)

$$Var(p_{u})=\frac{2l(s^{2}+2s+2)+m(s^{2}+3s+2)+n(s^{2}+s)+2v}{4{\left[l(s+2)+m(s+1)+ns+v\right]}^{2}}p(1-p)$$

$$Cov(p_{a},p_{u})=\frac{lr(s+2)+m(r+1)(s+1)+ns(r+2)}{\left[lr+m(r+1)+n(r+2)+u\right]\left[l(s+2)+m(s+1)+ns+v\right]}p(1-p),$$

And *p *is the estimated average allele frequency of all subjects in the data. For our final data, *r *= 2; *s *= 0; *l *= 140; *m *= 56; *n *= 2, and *u *= *v *= 200.

### In the presence of population stratification

In the situation of population stratification, we suggest an approach to adjust the genotype data using principal components before the above procedures are applied. Unfortunately, the RA data was simulated without a population stratification effect, therefore we only give brief idea of this method here. The rationale of this approach is that across the genome there should be a consistent pattern among allele frequency differences, and that pattern is summarized by principal components to which many markers contribute. We sketch the procedures below. Details may be found in Price et al. [3]. First, pick founders from each family and all unrelated case-controls. Denote the genotype at the *i*^th ^locus for *j*^th ^individual by *g*_*ij*_, *i *= 1,..., *M *and *j *= 1,..., *N*. Let $u_{i}=\frac{1}{N}\sum_{j=1}^{N}{\text{g}}_{\text{ij}}$ be the sample mean for *i*^th ^locus and *X *= (*x*_*ij*_) the matrix normalized by subtracting *u*_*i *_from each row and dividing by $\sqrt{\frac{1}{2}u_{i}(1-u_{i})}$. Second, compute the estimated covariance matrix of all markers $\psi_{M\times M}=\frac{1}{N-1}XX^{T}$, and list the first *k *largest eigenvalues *λ*_1_,..., *λ*_*k *_with corresponding eigenvectors *v*_1_,..., *v*_*k *_The *l*^th ^eigenvector *v*_*l *_= (*v*_*l*1_,..., *v*_*lM*_) gives the *l*^th ^principal component as $v_{l}\cdot g=(v_{l1},...,v_{lM})\cdot (g_{1},...,g_{M})=\sum_{i=1}^{M}v_{li}g_{i}$. Finally, regress genotypes on the markers by $g_{ij,update}=g_{ij}-\sum_{l=1}^{k}v_{li}\sum_{s=1}^{M}v_{ls}g_{sj}$, where $\sum_{s=1}^{M}v_{ls}g_{sj}$ is the regression coefficient for *l*^th ^marker and *j*^th ^individual.

## Results

Because population stratification was not simulated in GAW15, we did not adjust the genotype data using principal component procedures. We directly applied the test to the 9187 SNPs, and identified four SNPs whose *p*-values are far less than the Bonferroni corrected *p*-value 0.05/9187 = 5.44 × 10^-6^. We used the software Haploview [4] to test the linkage disequilibrium pattern among them. The *D*' scores among SNP6-152, SNP6-153, and SNP6-154 are above 0.93, suggesting strong LD, and the *D*' between SNP6-155 and the rest was less than 0.38. Next, we applied a case-control chi-square test to the unrelated 200 cases and controls, and a family-based test (transmission-disequilibrium test, or TDT) to the family data. As a comparison, we also applied our test *zfam *only to the family data. All the test results were consistent, and are summarized in Table 1. The squares of the new test value *z *are strictly larger than the square sum of the corresponding chi-square test and TDT. For the family data, the value of our statistic *zfam *is also bigger than the value of TDT test statistic. These suggest that the proposed combined test has improved power. Also, as expected, the values of test statistic *z *are much larger than the test statistic *zfam*, which is restricted only to families, because more information from the unrelated case-control sample is used.

**Table 1:** The most significant SNPs out of the total 9187 markers and their test values with associated *p*-values before Bonferroni correction.

SNP	Location (cM)	test *z*	*p*_*z*_	*χ*^2^	*p*_*x*2_	family *z*_*fam*_	*p*_*fam*_	TDT	*p*_*TDT*_
SNP6-152	49.4300	16.10	0	92.93	0	12.31	0	7.57	1.87 × 10^-14^
SNP6-153	49.4606	24.07	0	276.53	0	16.49	0	10.52	0
SNP6-154	49.4662	23.26	0	225.80	0	16.68	0	10.06	0
SNP6-155	49.6216	10.30	0	57.68	3.09 × 10^-14^	6.60	2.06 × 10^-11^	3.88	5.22 × 10^-5^

The type I errors of the proposed test are reasonable and comparable to the other two tests, which are listed in Table 2. At the significance level *α *= 0.05, we observed 483 SNPs with *p*-values less than 0.05, giving a slightly higher type I error rate of 0.0525, which might be caused by correlation with disease loci. Thus, we excluded all the 674 SNPs on chromosome 6, and then observed 433 SNPs with *p*-value less than 0.05, with a corresponding type I error of 0.0508 (Table 2). Next, we applied our test to the dense map of chromosome 6, and got 56 significant SNPs whose *p*-values are less than the Bonferroni corrected *p*-value 0.05/(17820 + 9187) = 1.85 × 10^-6^. In particular, the markers 3439, 3442, 3437, 3436, 3440, 3430, and 3426 have the largest test value. Together with the LD patterns from Haploview, we conclude that the most likely interval for a major gene is between 49.4262 cM and 49.5184 cM on chromosome 6.

**Table 2:** Type I error rates of different tests for all markers except those on chromosome 6.

Test	Type I error
Combined *z *test	0.0508
Family *z*_*fam*_	0.0507
Case/control *χ*^2^	0.0509
TDT	0.0506

## Discussion

Under the assumption of Hardy-Weinberg equilibrium, the proposed approach has improved power by combining families of different structures with unrelated subjects, and it also give a potential way to resolve the issue of population stratification. Compared with the traditional TDT test, the proposed test can combine all the available families and may have better power than the TDT because the TDT excludes a certain proportion of families. Under the assumption of no population stratification and low disease prevalence in parents, another simpler test that Risch and Teng describe is to regard all parents from families as unaffected, with the remainder of this test being the same as ours [2]. However, when we carried out this test on the RA data, it led to an inflated type I error rate. At the significance level *α *= 0.05, the type I error rate reached 0.055. On the other hand, our new proposed test might lose power without the random mating assumption.

Recently Epstein et al. [5] described a likelihood-based approach for combining triads and unrelated subjects, but it requires further work to combine families of different structures. Li et al. [6] also published another likelihood-based approach using hidden Markov model of affected sibling pairs. However, their approaches can not deal with the issue of population stratification. We proposed a principal-component based approach to resolve this, and will test the performance of adjusting population stratification procedure elsewhere.

## Competing interests

The author(s) declare that they have no competing interests.
